# Comparison of Different Polarization Sensitive Second Harmonic Generation Imaging Techniques

**DOI:** 10.3390/mps2020049

**Published:** 2019-06-07

**Authors:** Mehdi Alizadeh, Masood Ghotbi, Pablo Loza-Alvarez, David Merino

**Affiliations:** 1 Department of Physics, University of Kurdistan, Sanandaj 66177-15175, Iran; alizadehmehdi65@gmail.com (M.A.); masood_gh49@yahoo.com (M.G.); 2 ICFO-Institut de Ciencies Fotoniques, The Barcelona Institute of Science and Technology, 08860 Barcelona, Spain; Pablo.Loza@icfo.eu; 3 UOC, Universitat Oberta de Catalunya, 08018 Barcelona, Spain

**Keywords:** medical and biological imaging, nonlinear microscopy, polarization, second harmonic generation

## Abstract

Polarization sensitive second harmonic generation (pSHG) microscopy is an imaging technique able to provide, in a non-invasive manner, information related to the molecular structure of second harmonic generation (SHG) active structures, many of which are commonly found in biological tissue. The process of acquiring this information by means of pSHG microscopy requires a scan of the sample using different polarizations of the excitation beam. This process can take considerable time in comparison with the dynamics of in vivo processes. Fortunately, single scan polarization sensitive second harmonic generation (SS-pSHG) microscopy has also been reported, and is able to generate the same information at a faster speed compared to pSHG. In this paper, the orientation of second harmonic active supramolecular assemblies in starch granules is obtained on by means of pSHG and SS-pSHG. These results are compared in the forward and backward directions, showing a good agreement in both techniques. This paper shows for the first time, to the best of the authors’ knowledge, data acquired using both techniques over the exact same sample and image plane, so that they can be compared pixel-to-pixel.

## 1. Introduction

Second harmonic generation (SHG) microscopy is a technique used for non-invasive high-resolution imaging of different kinds of biological samples [1]. The technique features some interesting advantages for in-vivo imaging and clinical applications. First, no exogenous contrast agents (such as fluorescence markers) are needed to generate the images. Second, it presents high tissue penetration as usually excitation of the signal is performed in infrared. Third, the technique presents intrinsic depth discrimination with low photodamage [2,3]. Furthermore, polarization sensitive second harmonic generation (pSHG) imaging can be used to provide information related to the molecular structure of the SHG active components in many different types of biological samples [2,3,4,5,6,7]. In particular, the technique has been successfully reported on tissue samples of myosin, collagen, microtubules, starch and cellulose [3,6,8,9,10]. 

Usually, to generate pSHG images, a complex acquisition strategy is used, since the generation of these images requires excitation of the sample using light polarized along several different polarization directions. In our study, the process of generating pSHG images uses excitation light aligned along 9 different polarizations directions in steps of 20° (from 0° to 180°), and takes around 1.5 min for the whole process to complete [7,9,10,11]. Due to this reason, the technique may not suitable to study as in vivo dynamic processes.

Single scan polarization sensitive second harmonic generation (SS-pSHG) microscopy has been proposed to provide molecular information of SHG active molecules from in vivo samples, at a faster rate than in the case of pSHG, allowing observation of dynamic processes in vivo [12]. In SS-pSHG, a single circularly polarized light beam is used for excitation, and the final images can be produced in order of magnitude, faster than in the case of pSHG [12,13]. Therefore, SS-pSHG has great potential for studying fast dynamics in in vivo biological structures.

In this study, the results acquired using pSHG and SS-pSHG from starch granules in the forward and backward directions are compared. Despite the complexity of the acquisition system, which will be introduced in the following sections, this study was able, for the first time to the best of the authors’ knowledge, to produce pSHG and SS-pSHG data obtained from the same sample and on the same plane for both techniques. The results obtained by means of the two different techniques, physically correspond with each other, and were interpreted as a way to validate SS-pSHG results by comparing them with those obtained using pSHG microscopy.

## 2. Material and Methods

### 2.1. Biophysical Models

Two different biophysical models have been used in order to calculate the results presented in this work—one corresponding to pSHG and the second to SS-pSHG. These models have been described in more detail in previous studies [3,5,9,13,14,15,16], and are briefly introduced here. 

For convenience, this subsection is divided in two parts, each corresponding to each of the techniques.

#### 2.1.1. pSHG Biophysical Model

**Figure 1:**
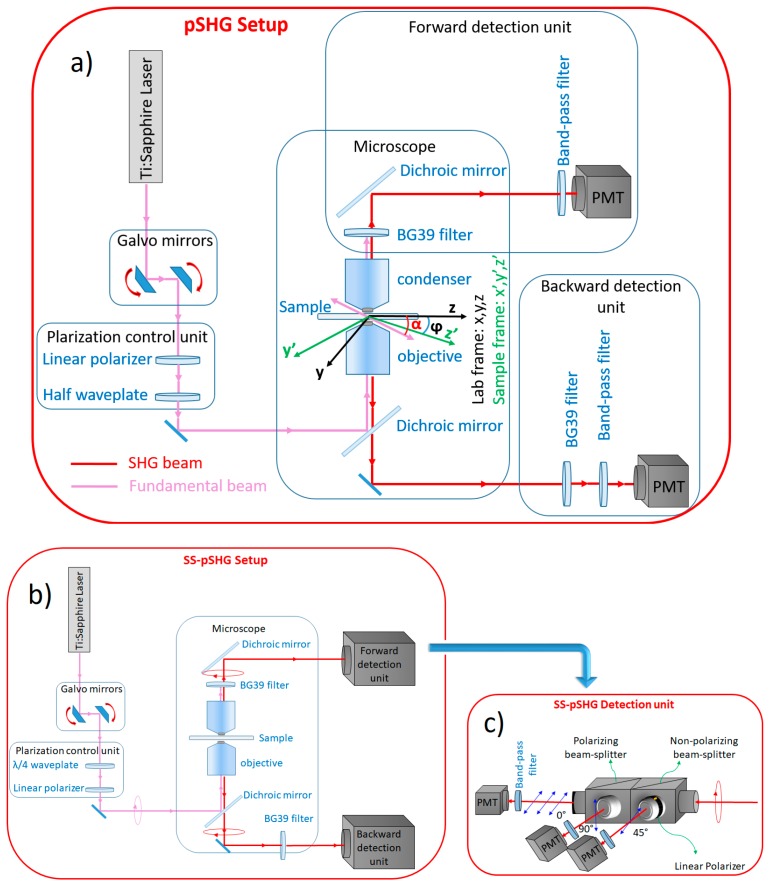
Schematic diagram of the optical setup: (**a**) The polarization sensitive second harmonic generation (pSHG) microscopy setup, where z’ is the orientation of the supramolecular assembly, which for the case of starch can be seen in Figure 2, *ϕ* is the angle of orientation of the assembly, and *α* is the orientation of the polarization of the excitation beam, (**b**) the single scan polarization sensitive second harmonic generation (SS-pSHG) microscopy setup and (**c**) the detection unit to collect the second harmonic generation (SHG) signal in three directions in the SS-pSHG setup. The same detection unit is used in the forward and backward directions.

Linearly polarized light is used to excite an SHG active supramolecular assembly with cylindrical symmetry of group C_6_, and assuming that the Kleinman symmetry condition is valid, the intensity of the SHG signal is generated, *I_SHG_*. This depends on the orientation angle of the supramolecular assembly, *φ*, and the polarization angle of the excitation beam, *α*. Figure 1a shows how these angles are defined in the imaging plane. According to this, the expression of *I_SHG_* is as follows [9]:

(1) $$I_{SHG}\left(\varphi ,\alpha \right)=a_{0}+a_{2}\;\cos\;2\left(\varphi -\alpha \right)+a_{4}\;\cos\;4\left(\varphi -\alpha \right),$$

where *a*_0_, *a*_2_ and *a*_4_ are coefficients related to the internal structure of the SHG active molecules.

By performing a one-dimensional Fourier transform (FT) on the polarization angle, *α*, to the previous expression, the following is obtained [9]:

(2) $$i\left(\Omega \right)=a_{0}\delta \left(0\right)+a_{2}\exp\left(i2\varphi \right)\delta \left(1-\Omega \right)+a_{4}\exp\left(i4\varphi \right)\delta \left(2-\Omega \right)+c.c.$$

where c.c. indicates the complex conjugate, and *Ω* is the Fourier variable corresponding to the polarization angle, α. 

From the second term of the Equation, the orientation of the supramolecular assembly, *φ*, can be calculated as follows:

(3) $$\varphi =\arg\left[a_{2}\exp\left(i2\varphi \right)\right]/2$$

Also, from the Equation, it is possible to calculate the orientation of the hyperpolarizability tensor dominant axis, which in some cases can be related to the helical pitch angle of the SHG active molecules, *θ_e_*, [9]:

(4) $$tan^{2}\theta_{e}=\frac{2}{\sqrt{\frac{a_{0}+a_{2}+a_{4}}{a_{0}-a_{2}+a_{4}}}}$$

It should be mentioned at this point that this model is accepted for the forward configuration, i.e., the case in which the SHG generated signal propagates in the same direction as the excitation beam. However, it has been described previously that the SHG signal has also been observed in the backwards direction in the case of starch. There are two different mechanisms that can explain this observation. First, would be scattering within the starch granule, since starch is highly scattering. Second, would be backwards phase matching. In this case, it has been shown that when the axial size of SHG active objects is smaller than λ_SHG_/10 (in our case ~40 nm), similar forward and backward phase matching occurs [17,18,19,20,21]. It has been described that the size of the semi-crystalline shells of amylopectin side chain clusters ~10 nm thick, which is well within this range of values [22,23].

#### 2.1.2. SS-pSHG Biophysical Model

In SS-pSHG imaging, excitation of the sample is achieved by means of a circularly polarized beam [12]. In this case, the SHG signal generated can be described as an elliptically polarized wave, with the long axis aligned along the direction of the orientation of the SHG active structure. In order to characterize this ellipse, the SHG signal can be divided into three different beams, and each propagated through an analyzer oriented along a different angle, *α*. The intensity of SHG signal detected after each of these analyzers depends on the supramolecular assembly orientation,φ, as follows [12]:

(5) $$I_{SHG}^{\alpha }\propto E_{0}^{2}\left(sin^{2}\left(\varphi -\alpha \right)+\frac{1}{4}{\left(\frac{d_{33}}{d_{15}}-1\right)}^{2}cos^{2}\left(\varphi -\alpha \right)\right)$$

where $E_{0}$ is the excitation electric field amplitude, $d_{33}$ and $d_{15}$ are related to the elements of the second order susceptibility tensor $\chi^{\left(2\right)}$ [12]. By setting α in each of the three analyzers to the values $\alpha_{1}=0{}^{\circ}$,$\alpha_{2}=45{}^{\circ}$ and $\alpha_{3}=90{}^{\circ}$, the $I_{SHG}^{0}$, $I_{SHG}^{45}$ and $I_{SHG}^{90}$ intensities respectively can be acquired from the Equation. From that equation, the value of the orientation of the polarization ellipse with respect to the lab axis, *φ*, can be obtained from the intensities $I_{SHG}^{0}$, $I_{SHG}^{45}$ and $I_{SHG}^{90}$, as:

(6) $$\varphi =\frac{1}{2}\tan^{-1}\left\{\frac{2I_{SHG}^{45}-I_{SHG}^{0}-I_{SHG}^{90}}{I_{SHG}^{0}-I_{SHG}^{90}}\right\}$$

In the case of starch, the anisotropy parameter can also be obtained from the polarization ellipse analysis as [12]:

(7) $$\frac{d_{33}}{d_{15}}=1+2\sqrt{\frac{I_{SHG}^{0}cos^{2}\left(\varphi \right)-I_{SHG}^{90}sin^{2}\left(\varphi \right)}{I_{SHG}^{90}cos^{2}\left(\varphi \right)-I_{SHG}^{0}sin^{2}\left(\varphi \right)}}$$

### 2.2. The Microscope Setup

In this work, the forward and backward pSHG and SS-pSHG images of biological samples have been acquired sequentially using the setups described in this section, without changing the sample. As in the previous case, we have divided this section into two parts—first, the setup of the pSHG experiment, and then the setup for SS-pSHG experiment. 

#### 2.2.1. pSHG Microscope Setup

The pSHG microscopy setup, which is shown in Figure 1a, has been fully described in previous studies [4,8,16]. A Kerr lens modelocked Ti:sapphire laser (MIRA 900f, Coherent, Santa Clara, CA, USA) was used as the excitation source. This source generates laser pulses at a central wavelength of 810 nm with a duration of 160 fs (measured at the sample plane) and a repetition rate of 76 MHz. The power of the beam was controlled by a neutral density wheel and the power was maintained in a range where no observable damage occurred for long imaging periods of time. The optical power on the sample plane was measured to be 35 mW, and was maintained at this level in all the experiments.

A pair of galvanometer (galvos) mirrors (Cambridge Technology, Bedford, MA, USA) was used as the y-z line scanning unit. A linear polarizer (ThorLabs, LPNIR050, Newton, NJ, USA) was placed after the galvos to reduce any introduced ellipticity. This element was followed by a zero order λ/2 wave plate (ThorLabs, WPH10M-808, Newton, NJ, USA) that was placed on a motorized rotational stage (AG-PR100, Newport Corporation, Irvine, CA, USA) to rotate the linear polarization at the sample plane. A lab-VIEW (National Instruments Corporation) interface program was written to control the raster scanning of the galvos and the data acquisition card. Finally, a short wave-pass dichroic beamsplitter (FF720-SDi01-25×36, Semrock Inc, Rochester, NY, USA) was introduced before the microscope objective. In order to focus the beam on the sample, an oil immersion 1.4 NA 60× objective (Plain Apo-Achromatic, Nikon, Japan) was used, and a 1.4 NA oil immersion condenser was used to collect the signal generated in the forward direction. The theoretical axial resolution of the system was ~1.0 µm [24]. Typical frame acquisition time for a single 512 × 512 pixels image was about ~1.5 s. The pixel dwell time of our system was ~6 µs.

A proper mount and detection unit was placed after the condenser in the forward direction. This unit contained a long wave pass dichroic beamsplitter (FF665-Di02-25×36, Semrock Inc, Rochester, NY, USA), a BG39 filter, a 15-nm full width at half maximum (FWHM) band-pass filter centered at 406 nm (FF01- 406/15-25, Semrock Inc, Rochester, NY, USA) and a photomultiplier tube (PMT) (H9305-04, Hamamatsu, France). In the backward direction, a similar detection unit was placed after the dichroic, including a band-pass filter and a PMT.

Nine different pSHG images were obtained by exciting the sample with the same number of different linear polarizations. The rotating half wave plate was used to change the polarization of the excitation beam reaching the sample. SHG images were obtained for the polarization values ranging from 0 to 180° at 20° steps, and then stored for post-processing. In an attempt to reduce the noise in the images, each polarization experiment was repeated four times and then averaged. Although each of the images was acquired in ~1.5 s, the overall acquisition time of a pSHG experiment was ~1.5 min.

#### 2.2.2. SS-pSHG Microscope Setup

The experimental setup for the SS-pSHG is shown in Figure 1b, which is based on that used for pSHG described in Section 2.2.1. The excitation beam was generated by the same pulsed laser source, although in this case it was operated at a central wavelength of 850 nm. It is noted that this change of wavelength only responds for a practical reason, given the availability in our lab of the different polarization elements needed for this setup (in other words, the technique is valid for any excitation wavelength if the adequate filters are used). In this experiment, a linear polarizer (ThorLabs, LPNIR050, Newton, NJ, USA) was also placed after the galvos, and then a zero-order quarter-wave retardation plate (WPQ10M-850, Thorlabs, Germany) was followed at the proper angle to create circular polarization. The beam was then reflected on a short wave pass dichroic beamsplitter (FF720-SDi01-25×36, Semrock Inc, Rochester, NY, USA). At this point, the polarization of the beam was checked to ensure the beam was circularly polarized. To do so, a linear polarizer was placed in front of a detector in the objective plane. This was monitored to ensure that changing the angle of the polarizer did not change the readings in the detector, ensuring circularly polarized light. Once circularly polarized light was ensured, the detector and polarizer were removed, and the beam was focused on the sample using an oil immersion objective (1.4 NA 60×). As in the previous case, the optical power on the sample plane was measured to be 35 mW, and was maintained at this level in all the experiments. 

The generated elliptically polarized SHG signal was collected both in the forward and backward directions. In the forward direction, the beam was guided through a dichroic mirror (FF552-Di02-25×36, Semrock Inc, Rochester, NY, USA) to a specially designed detector mount shown in Figure 1c. The mount divided the signal into three paths by using a 50:50 non-polarizing beam-splitter cube (10BC17MB.1, Newport, Irvine, CA, USA). Half of the signal was propagated through a linear polarizer (10LP-Vis-B, Newport, Irvine, CA, USA) at 45°. The other half is sent to a second beam-splitter cube, in this case a polarizing one (10FC16PB.3, Newport, Irvine, CA, USA), which separates light polarized at 0° and 90°. As a result of this manipulation, the SHG signal was divided into three components, each corresponding to light polarized along 0°, 45° and 90°. Each of these components was detected by means of a PMT (H9305-03, Hamamatsu, Japan) after being filtered by a 10 nm FWHM band-pass filters centered at 427 nm (FF01-427/10-25, Semrock, Rochester, NY, USA). In order to reduce the noise in the images, the experiment was repeated five times for each polarization and then averaged. The acquisition time for each SS-pSHG experiment was ~7.5 s.

### 2.3. Starch Sample

**Figure 2:**
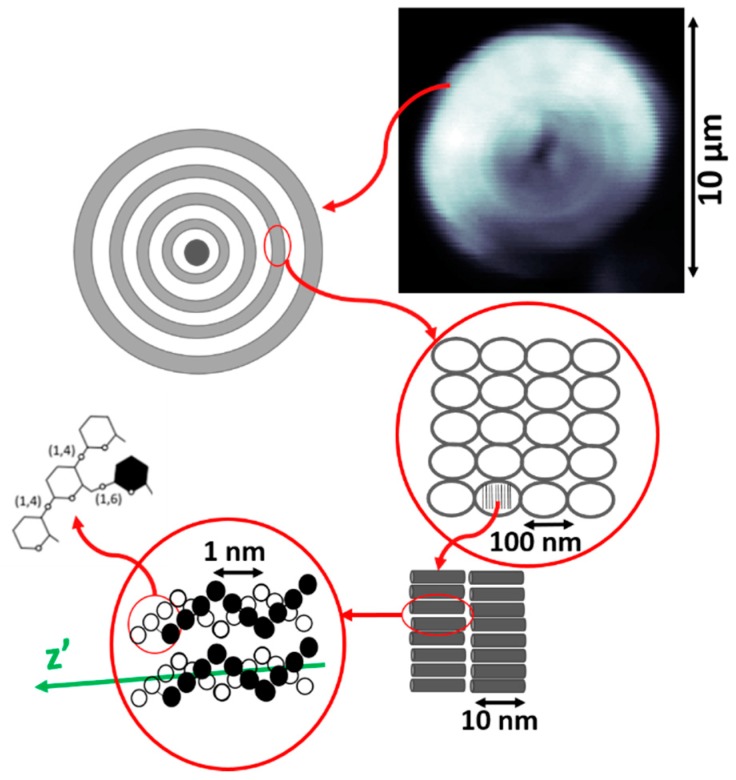
Schematic drawing of the internal structure of a starch granule, where z is the axis along which the cylindrical supramolecular assembly is oriented [27].

To perform the experiments that are described in this manuscript, samples of starch granules have been used. Starch granules are made of microscopic complex networks of amylose and amylopectin, organized in alternating concentric 120–400 nm thick amorphous and semi-crystalline domains, known as growth rings, which can be seen in Figure 2 [22,25,26,27]. These semi-crystalline layers consist of ordered regions comprising double helical structures formed by short amylopectin branches, and produce a strong second-order nonlinear optical response, being one of the brightest SHG converters found in nature [8,11,28,29,30,31,32]. 

In order to prepare the samples used in this experiment, a few starch granules were placed on a microscope slide along with a drop of distilled water. The sample was protected by a cover slip that was fixed to the slide. In order to ensure that the orientation of the amylopectin branches are aligned with the imaging plane of the microscope, the images of the sample were obtained focusing on the central plane of the granule [12].

## 3. Results

In this section, the results of the data obtained from starch granules using pSHG and SS-pSHG are compared. The section is divided in three subsections: One to describe the results obtained by using pSHG in the forward and backward directions; a second to compare the results obtained by pSHG and SS-pSHG both in the forward direction; a third subsection to compare the results obtained by pSHG and SS-pSHG in the backward direction. It should be clarified that the images in each experiment are from the same sample at the same imaging plane. However, different experiments show different samples and image planes.

### 3.1. Forward pSHG vs Backward pSHG

**Figure 3:**
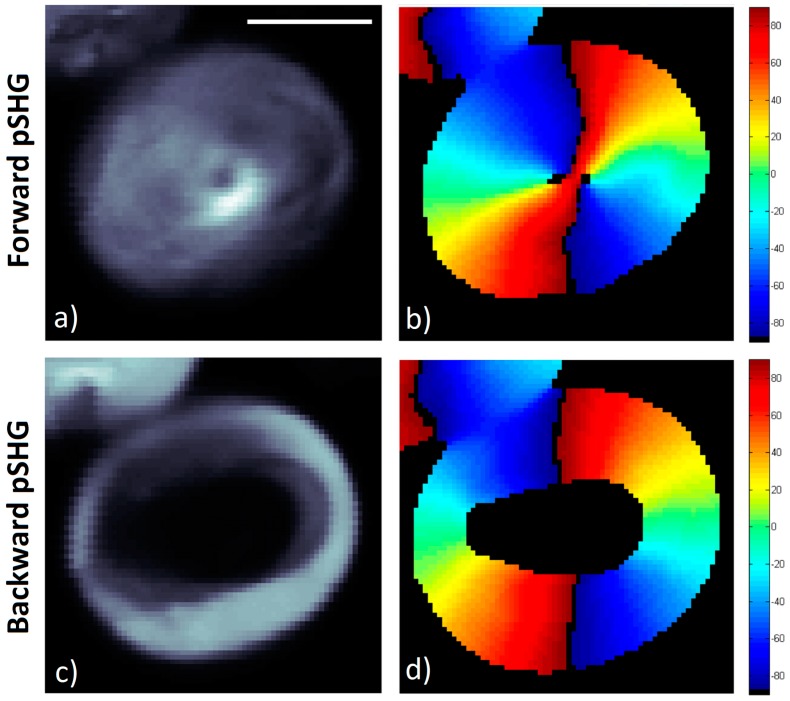
Forward and backward pSHG images acquired from starch granules. The images in both directions are obtained simultaneously in the same scan. Images are 70 × 70 pixels, with a pixel size of ~195 nm. Scale bar is 5 µm, and the acquisition time for these images was ~1.5 min. (**a**) Average intensity of the SHG images generated by 9 linear polarized excitation beams in forward direction. (**b**) Amylopectin supramolecular assembly orientations, *φ*, calculated from data in (a). (**c**) Average of the 9 intensity SHG images generated in the backward direction. (**d**) Assembly orientation values, *φ*, calculated from data in (**c**).

Figure 3 shows representative images of the pSHG data obtained from the same plane of a starch granule in the forward and backward directions simultaneously. The setup shown in Figure 1a was used to acquire these images, as described previously.

The average of the different SHG intensity images generated for the different excitation polarization directions in the forward direction is shown in Figure 3a. The results of the orientation of the SHG active amylopectin supramolecular assembly is shown in Figure 3b, *φ,* calculated using the Equation (3) after post processing of the acquired data. In the image, pixels with low signal to noise ratio (SNR) appear in black since they have been rejected from the image following the criteria detailed in previous reports [7]. In other words, a mask has been generated selecting the pixels that, after processing, show enough SNR, to confirm that these are the pixels within the granule, as opposed to those outside, where weak or no SHG signal is observed.

Figure 3c shows the average of the SHG signal intensity images for the same starch granule shown in Figure 3a, although the SHG signal has been collected in the backward direction. It is interesting to note that the SHG signal in the backward direction appears more intense in the outer layers of the starch granule than in the inner ones, as has been reported previously [17,33]. As previously discussed, differences in the SHG signal directionality may be explained by means of differences in domain sizes that may generate a SHG signal in the backwards direction [17,18,19,21] or by the SHG generated in the forward direction, which is then elastically scattered back. Due to this decrease in signal in the inner layers of the granule, these pixels have been rejected, and the resulting SNR mask forms a ring.

Despite the fact that the biophysical model described in Section 2.1 has been developed and considering the SHG signal is collected following the direction of the excitation light (in this experiment, in the forward direction), the data in Figure 3c has been processed using the same algorithm, and the results are shown in Figure 3d.

**Figure 4:**
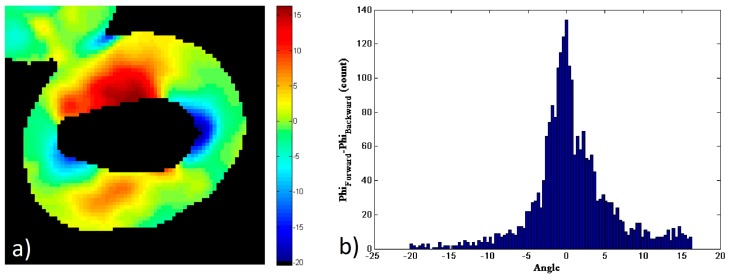
(**a**) Subtraction of the orientation of the amylopectin supramolecular assembly values, *φ_s_*, calculated using data acquired in the forward, Figure 3b, and backward, Figure 3d, directions. (**b**) Histogram of *φ_s_* values in (**a**).

Figure 4 shows the comparison of the data in the forward and backward directions for this particular image of a starch granule. To generate this figure, a new mask of the data has been generated, for only the pixels that have enough SNR on both the forward and backward directions. Figure 4a shows the pixel-to-pixel subtraction of the amylopectin supramolecular assembly orientation, *φ_s_*, obtained from the forward and backward pSHG images shown in Figure 3:

(8) $$\varphi_{s}=\varphi_{Forward}-\varphi_{Backward}$$

Figure 4b shows the histogram of the values shown in Figure 4a. The results show that the values of *φ_s_* are distributed between −20° and 17°, although most of these are close to 0°. The FWHM of the histogram is ~4.9° and 52% of the pixels fall in the FWHM peak of the histogram, i.e. $\left|\varphi_{s}\right|<2.5{}^{\circ}$. These results show that the *φ* values obtained using forward and backward pSHG are in a good agreement. This is in itself an interesting result, since the biophysical model of pSHG imaging presented in this manuscript is conceived for the forward imaging configuration, but the results obtained in the backward direction seem also valid. Furthermore, this result allows the characterization of the expected error when the model is used in the backward direction configuration.

### 3.2. Forward pSHG vs Forward SS-pSHG

In this subsection, the results obtained using pSHG and SS-pSHG in the forward direction are compared.

In this experiment, images from the starch granule were performed using the pSHG setup shown in Figure 1a. Then, the appropriate changes in the setup were introduced to transform the acquisition system to that in Figure 1b. These included changing the wavelength of the excitation laser, replacing the half-wave plate that was mounted after the galvanometer scanners with a quarter-wave plate, change the microscopy dichroic cubes to match the new wavelength, and finally, putting the detection module in the forward direction. The setup was optimized to perform these changes in a matter of a couple of minutes, minimizing any possible effects of motion or drift in the sample.

**Figure 5:**
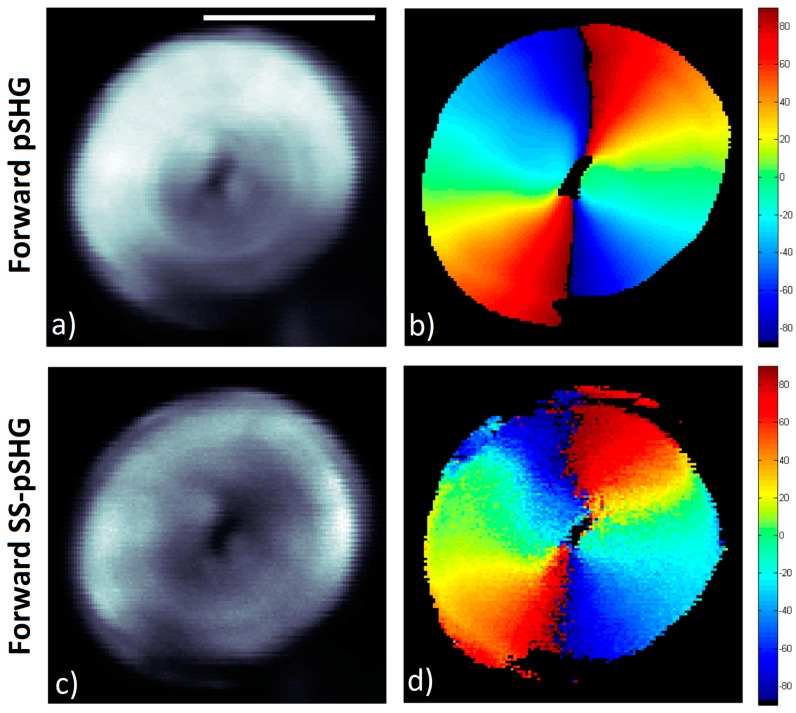
pSHG and SS-pSHG images acquired from starch granules in the forward direction. Images are 135 × 115 pixels, with pixel size of ~ 86 nm. Scale bar is 5 µm. (**a**) Average of forward SHG intensity images acquired using 9 different linear polarization of the excitation beam. Acquisition time for this pSHG image was 1.5 min. (**b**) Amylopectin supramolecular assembly orientation, *φ*, calculated from data in (**a**). (**c**) Average of forward SHG intensity images acquired from 0°,45° and 90° channels in SS-pSHG. Acquisition time for this image was ~7.5 s. (**d**) Assembly orientation values, *φ*, calculated from data in (**c**).

The results obtained are shown in Figure 5. Similar to the previous section, Figure 5a,c show the average SHG intensity images of the data acquired from the same starch granule using pSHG and SS-pSHG respectively. As discussed previously, in the case of pSHG, this image is generated from averaging the SHG images acquired by changing the linear polarization angle of the excitation beam. In the case of the SS-pSHG, Figure 5c is generated by averaging the three images obtained changing the orientation of the linear polarizer (along 0°, 45° and 90°).

Figure 5b,d shows the orientations of the amylopectin supramolecular assembly for each pixel, calculated for the pSHG and SS-pSHG data respectively. Both techniques show the radial structure of the starch granule, although data seems noisier in the case of SS-pSHG. This is likely due to the fact that pSHG images are generated using 9 images, with excitation polarization oriented along 9 different directions. This means that 9 different scans where performed to acquire each of the images. As previously discussed, these experiments were averaged usually 4 times to reduce this noise. In the case of SS-pSHG, the three SHG images were generated from just one scan. Even though the experiment was averaged 5 times, the overall dwell pixel time was 7 times lower in the case of SS-pSHG compared to pSHG. In other words, the decrease in the number of acquisition exposures in SS-pSHG imaging with respect to pSHG has an impact in the noise of the final data. However, this reduction in experiment repetitions and the fact that the polarization is not scanned throughout the experiment, the time needed to generate the data is reduced from ~1.5 min in the case of pSHG to ~7.5 s in the case of SS-pSHG.

**Figure 6:**
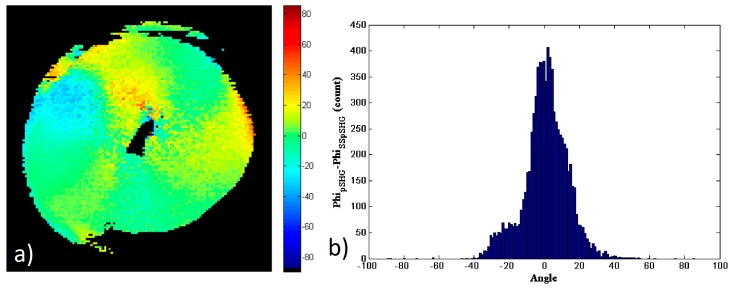
(**a**) Subtraction of the *φ* values obtained by means of pSHG and SS-pSHG in the forward direction shown in Figure 4b,d. (**b**) Histogram of *φ_s_* values shown in (**a**).

Similar to the comparison of data in the forward and backward directions of the pSHG experiment, the subtraction of Figure 5b,d has been calculated to investigate the similarity of the results obtained by means of pSHG and SS-pSHG in the forward direction. These results are shown in Figure 6a, and the corresponding histogram of this subtraction image is shown in Figure 6b. The values of the difference of the pSHG and SS-pSHG values show a peak around 0°. In this case, the FWHM of the histogram is ~19° and ~66% of the pixels which have $\left|\varphi_{s}\right|<9.5{}^{\circ}$. The increase in the FWHM in this experiment is related to the increased noise observed in the SS-pSHG images with respect to those acquired by means of pSHG. These results allow to the validation of those related to the orientation of the amylopectin supramolecular assemblies obtained by means of SS-pSHG, as they are in good agreement with those acquired by means of pSHG.

### 3.3. Backward pSHG vs Backward SS-pSHG

**Figure 7:**
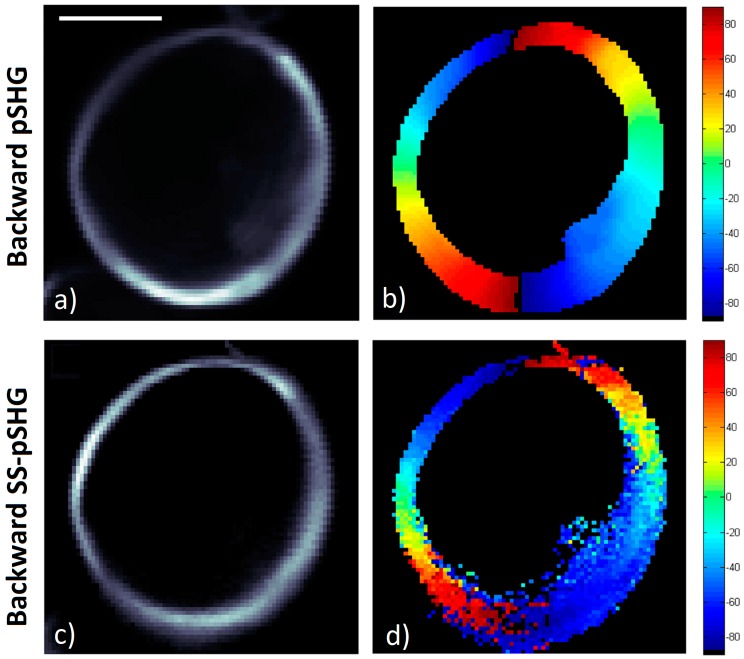
pSHG and SS-pSHG images acquired from starch granules in the backward direction. Images are 70 × 70 pixels, with a pixel size of ~195 nm. Scale bar is 5 µm. (**a**) Average of backward SHG intensity images acquired using 9 different linear polarizations of the excitation beam. Acquisition time for this pSHG image was 1.5 min. (**b**) Supramolecular assembly orientations, *φ*, calculated from data in (**a**). (**c**) Average of the backward SHG intensity images acquired from 0°, 45° and 90° channels in SS-pSHG. Acquisition time for this SS-pSHG image was ~7.5 s. (**d**) Assembly orientation values, *φ*, values calculated from data in (**c**).

In this section, the results of the amylopectin orientation values in a starch granule, *φ*, obtained in the backward direction by means of pSHG and SS-pSHG are compared. Similar to the previous section, Figure 7a,c show the average pSHG and SS-pSHG intensity images. As in the case of pSHG backward images, the images show that SHG intensity in the backward direction is stronger in the outer part of the granule structure, and this is visible for both the pSHG and SS-pSHG images.

Figure 7b,d show the *φ* values calculated from the data in Figure 7a,c, respectively. The radial structure of the starch granule is once again recognizable in both Figure 7b,d. 

**Figure 8:**
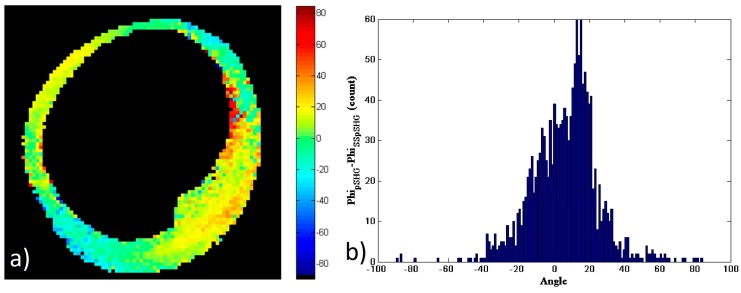
(**a**) Subtraction of the *φ* values obtained by means of pSHG and SS-pSHG in the backward direction shown in Figure 6b,d. (**b**) The histogram of *φ_s_* values.

As in the previous subsections, a new mask of the data has been generated and the study considered only the pixels that had enough SNR in both of the images in Figure 7b,d to calculate the subtraction of the results, *φ_s_*, shown in Figure 8a. Figure 8b shows the histogram of the values in the image (a). As in the previous cases, the histogram shows a peak approximately zero, and the FWHM of the histogram is ~30°, with approximately 66% of the pixels showing values of $\left|\varphi_{s}\right|<15{}^{\circ}$. 

These results show that the orientation of the amylopectin molecule measured by means of pSHG microscopy in the backward direction are in agreement with those acquired using SS-pSHG.

## 4. Discussion

In this study, the results of the orientation of SHG active amylopectin supramolecular assembly that forms starch granules, *φ*, obtained using pSHG and also SS-pSHG microscopy techniques both in the forward and backward directions have been compared. 

Two different setups of the image acquisition system were designed in a way that allowed easily transitioning from one to the next in a matter of a few minutes. This allowed the reduction of motion or drifting of the sample, and therefore the different images could be compared pixel-to-pixel to compare the differences in the results. 

With this setup, the different SHG imaging modalities have been compared. First, the forward and backward pSHG images were taken simultaneously, and therefore the results could be compared. These show that, although the biophysical model is designed for a forward emitting SHG structure, the data processing algorithm can be also used in the backward direction. Furthermore, although differences in the SHG intensity images were observed between the forward and backward generated images, the results of the algorithm corresponded amongst the two different configurations.

In a second experiment, after pSHG image acquisition, the setup was changed to SS-pSHG setup in the forward direction. In this case, simultaneous forward and backward SS-pSHG images could not be acquired. SS-pSHG needs three PMT detectors working simultaneously to generate the data. The hardware used only allowed three detectors to work simultaneously, and therefore forward and backward SS-pSHG imaging could only be performed sequentially. For that reason, a third experiment to compare pSHG and SS-pSHG in the backward direction was performed.

After post processing of the experimental data acquired, the *φ* values calculated corresponded in the forward and backward pSHG and forward and backward SS-pSHG. To compare these results, data obtained using the different modalities in each experiment were compared by subtracting the orientation values in each modality. A histogram of these subtractions, *φ_s_*, was generated. 

In the case of forward and backward pSHG, the FWHM of the histogram observed was approximately 4.9°, and 52% of the values decrease in the interval of FWHM, although −20°< *φ_s_* <17° for all of the pixel. In the case of forward pSHG compared to the forward SS-pSHG technique, the FWHM of the histogram of *φ_s_* values was approximately 19° and 66% of the pixels that have *φ_s_* values in the interval of the FWHM. The higher error observed in these results can be related to the noise in the SS-pSHG images. This noise may be related to the fact that SS-pSHG data is generated using a limited number of SHG images compared to pSHG. This reduces acquisition time, and makes the technique more suitable for the observation of dynamic processes. However, the results obtained are noisier, and present higher experimental error. 

Finally, the results obtained by backward pSHG and backward SS-pSHG were compared. The comparison of the results show a FWHM of approximately 30° for histogram of *φ_s_* values, and in this case, 66% of the pixels have *φ_s_* values in the interval of FWHM. The higher error in the results may be again related to a lower SNR in the intensity of the SHG signal in the backward compared to the forward direction. Despite the higher noise, which is nonetheless expected, the results show that, although the biophysical models in both techniques were designed for the forward imaging configuration, it is possible to apply the same processing algorithm to the data acquired in the backward direction in both of the techniques. This setup may be of interest in biomedical applications where it is not possible to analyze the signal in the forward configuration and at the same time, a fast measurement is needed. This is the case of the application of SHG to study corneal collagen in vivo. Also related to this application, the option of increasing the excitation power to reduce noise was dismissed in these experiments, as a way to consider the limitations that usually are present when dealing with biological tissue.
